# Establishment of a risk classifier to predict the in-hospital death risk of nosocomial fungal infections in cancer patients

**DOI:** 10.1186/s12879-023-08447-x

**Published:** 2023-07-17

**Authors:** Ruoxuan Wang, Aimin Jiang, Rui Zhang, Chuchu Shi, Qianqian Ding, Shihan Liu, Fumei Zhao, Yuyan Ma, Junhui Liu, Xiao Fu, Xuan Liang, Zhiping Ruan, Yu Yao, Tao Tian

**Affiliations:** 1 Department of Medical Oncology, The First Affiliated Hospital of Xi’an Jiaotong University, No. 277 Yanta West Road, Xi’an, Shaanxi 710061 People’s Republic of China; 2 Department of Medical Oncology, Baoji Traditional Chinese Medicine Hospital, No.43 Baofu Road, Baoji, Shaanxi 721001 People’s Republic of China; 3 Department of Clinical Laboratory, The First Affiliated Hospital of Xi’an Jiaotong University, No. 277 Yanta West Road, Xi’an, Shaanxi 710061 People’s Republic of China

**Keywords:** Cancer patients, Nosocomial infections, Fungal infections, Risk factors, In-hospital mortality, Nomograms

## Abstract

**Background:**

Patients with malignancy are at a higher risk of developing nosocomial infections. However, limited studies investigated the clinical features and prognostic factors of nosocomial infections due to fungi in cancer patients. Herein, this study aims to investigate the clinical characteristics of in-hospital fungal infections and develop a nomogram to predict the risk of in-hospital death during fungal infection of hospitalized cancer patients.

**Methods:**

This retrospective observational study enrolled cancer patients who experienced in-hospital fungal infections between September 2013 and September 2021. Univariate and multivariate logistic regression analyses were performed to identify independent predictors of in-hospital mortality. Variables demonstrating significant statistical differences in the multivariate analysis were utilized to construct a nomogram for personalized prediction of in-hospital death risk associated with nosocomial fungal infections. The predictive performance of the nomogram was evaluated using receiver operating characteristic (ROC) curves, calibration curves, and decision curve analysis.

**Results:**

A total of 216 participants were included in the study, of which 57 experienced in-hospital death. *C.albicans* was identified as the most prevalent fungal species (68.0%). Respiratory infection accounted for the highest proportion of fungal infections (59.0%), followed by intra-abdominal infection (8.8%). The multivariate regression analysis revealed that Eastern Cooperative Oncology Group Performance Status (ECOG-PS) 3–4 (odds ratio [OR] = 6.08, 95% confidence interval [CI]: 2.04–18.12), pulmonary metastases (OR = 2.76, 95%CI: 1.11–6.85), thrombocytopenia (OR = 2.58, 95%CI: 1.21–5.47), hypoalbuminemia (OR = 2.44, 95%CI: 1.22–4.90), and mechanical ventilation (OR = 2.64, 95%CI: 1.03–6.73) were independent risk factors of in-hospital death. A nomogram based on the identified risk factors was developed to predict the individual probability of in-hospital mortality. The nomogram demonstrated satisfactory performance in terms of classification ability (area under the curve [AUC]: 0.759), calibration ability, and net clinical benefit.

**Conclusions:**

Fungi-related nosocomial infections are prevalent among cancer patients and are associated with poor prognosis. The constructed nomogram provides an invaluable tool for oncologists, enabling them to make timely and informed clinical decisions that offer substantial net clinical benefit to patients.

## Introduction

Currently, cancer has emerged as a global public health concern that demands significant attention. Due to the presence of malignancy and frequent anti-tumor therapy, cancer patients are more susceptible to acquiring nosocomial infections [1, 2]. Additionally, this population often undergoes invasive procedures, including surgery, tissue biopsy, and catheter placement, which significantly increase their risk of acquiring nosocomial infections [3, 4]. Therefore, nosocomial infections have become one of the most common complications in oncological patients. Once a severe infection occurs, it undoubtedly hampers the initiation of anti-tumor treatment, prolongs hospitalization, increases healthcare-related burdens, and, in severe cases, can result in patient mortality. Consequently, infections have emerged as the primary non-cancer cause of death among cancer patients [5, 6]. Therefore, it is imperative for clinicians to comprehensively comprehend the clinical characteristics and prognostic factors associated with nosocomial infections in cancer patients.

In recent decades, the clinical features, microbiological distribution, and prognostic factors of in-hospital bacterial infections have been well documented [1, 2, 7–11]. Furthermore, pertinent guidelines have been published to provide guidance on managing bacterial infections acquired during hospitalization in this particular population [12–14]. It is worth noting that recent studies have highlighted the significant role of fungi as the primary causative pathogens of nosocomial infections in cancer patients [15–17]. In our previous study, we conducted a thorough investigation into the microbiological distribution of nosocomial infections in cancer patients. Our findings revealed that fungi constituted 11.4% of the identified causative pathogens [16]. Fungi are unique in that they do not produce endotoxins and exotoxins [18]. However, patients with malignancies face a heightened risk of fungal infections due to compromised immune function, which is associated with unfavorable clinical outcomes [19, 20]. In this context, invasive fungal disease (IFD) will occur in severe cases [21]. Furthermore, the prolonged administration of antifungal therapy, coupled with malnutrition and secondary infections, will heighten the risk of in-hospital mortality among these patients [4].

It is well known that there is a lack of comprehensive studies focusing on the clinical characteristics and prognostic factors of nosocomial fungal infections in cancer patients. Most importantly, no risk stratification system was developed to predict the in-hospital mortality rate of nosocomial fungal infections in cancer patients. In this premier, we conducted this retrospective study to explore the clinical features and prognostic factors of nosocomial fungal infections in this vulnerable population. Besides, we also aimed to construct a novel predictive model to robustly predict their risk of in-hospital death during nosocomial fungal infections, thus providing valuable guidance for clinical decision-making.

## Methods

### Study design

This retrospective observational study was conducted at the First Affiliated Hospital of Xi’an Jiaotong University in China from September 2013 to September 2021. This hospital, located in northwest China, is affiliated with a university and serves as a regional medical center. It houses a dedicated cancer treatment center that offers a comprehensive range of anti-tumor treatments, including surgery, chemotherapy, radiotherapy, and immunotherapy. This study enrolled patients who fulfilled the following criteria: (1) age 18 years and above; (2) laboratory test results indicative of fungal infection diagnosis; (3) confirmed presence of solid tumors through histological or cytological pathology; (4) diagnosis of nosocomial fungal infections during hospitalization; and (5) availability of complete electronic medical records (EMR) for the patients. Patients younger than 18 years old with incomplete EMR were excluded from the study. This study was approved by the Ethics Committee of the First Affiliated Hospital of Xi’an Jiaotong University (No: XJTU1AF2020LSK-049) and conducted in accordance with the principles outlined in the Declaration of Helsinki.

### Data collection

All data were extracted from the EMR and recorded in Microsoft Excel. The demographic data collected in this study encompassed age, gender, and smoking history. Cancer related variables included Eastern Cooperative Oncology Group-Performance Status (ECOG-PS), tumor type, TNM staging, sites of distant metastases, Charlson comorbidity index (CCI), anti-tumor therapy (including but not limited to surgery, chemotherapy, immune checkpoint inhibitor therapy, and radiotherapy) within 30 days, corticosteroid therapy in the past 30 days, granulocyte colony-stimulating factor (G-CSF) usage in the past 30 days and invasive procedures in the last one month. Simultaneously, we collected information pertaining to the infection, which encompassed the primary site of infection, fungal species, the coexistence of bacterial infection, initiation time and types of intravenous antifungal drugs, presence of fever, and antibiotic therapy received within the preceding 30 days. Additional variables, including admission to the intensive care unit (ICU), mechanical ventilation, and clinical outcomes following fungal infection (in-hospital mortality or discharge), were also documented. Furthermore, the most unfavorable outcomes from laboratory tests conducted prior to the diagnosis of fungal infection were recorded, encompassing blood routine tests, serum albumin levels, and serum electrolyte levels.

#### Definition

Nosocomial fungal infections were defined based on the following criteria: (a) positive culture of one or more fungal pathogens from clinical specimens (> 48 h after hospital admission), excluding cases of specimen contamination; and (b) confirmed diagnosis of fungal infections in the EMR by qualified physicians. Otherwise, the case was considered community-onset [22–24]. Once patients were suspected of having a fungal infection, various clinical samples, such as sputum, urine, and blood cultures, were collected. Fever was defined as either a single axillary temperature ≥ 38.3 ℃ or two or more temperatures ≥ 38.0 ℃within a 12-hour period [25]. Shock was determined by a systolic blood pressure below 90 mmHg, which did not improve with fluid therapy and/or vasoactive drugs [24].

### Study outcome

In the present study, we mainly focused on in-hospital fatality caused by nosocomial fungal infections. In-hospital mortality rate estimation did not include other causes of death, such as malignant tumors or unrelated factors.

### Statistical analysis

Fisher exact test or Chi-square test was used to compare the proportional differences of categorical variables. We used an independent sample *t-*test or non-parametric rank sum test to compare the differences of continuous variables. The independent influencing factors of in-hospital mortality were determined using univariate and multivariate logistic regression analyses. In the multivariate analysis, variables with a significant association (*P* value < 0.05) were selected to construct a nomogram for predicting the probability of in-hospital mortality due to fungal infection. The predictive performance of the nomogram was evaluated using receiver operating characteristics (ROC) curve analysis, calibration curve analysis, and decision curve analysis (DCA). All statistical analyses were performed using R software (version 4.1.3) for Windows 64.0.

## Results

### The essential characteristics of the participants

**Fig. 1:**
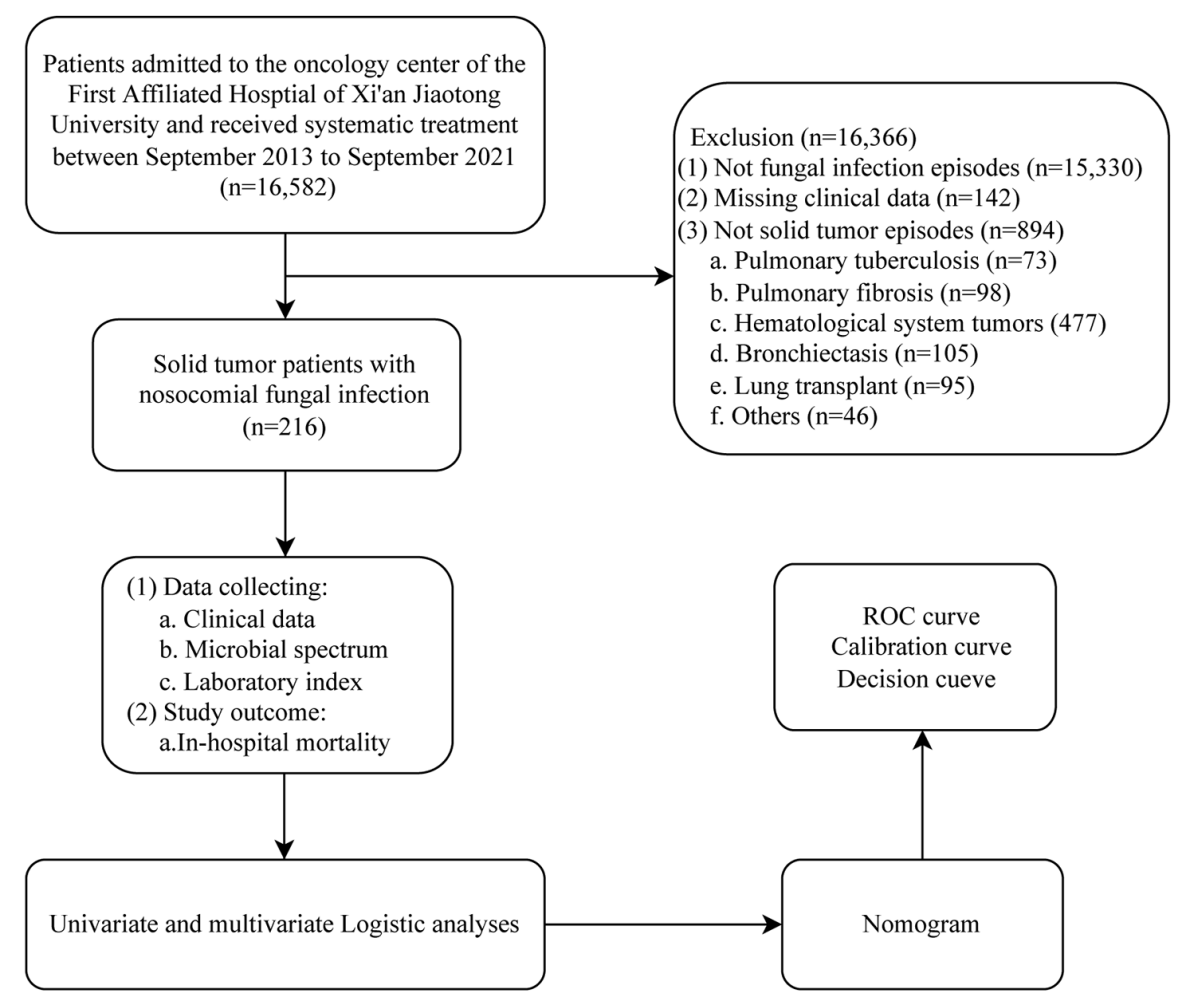
Flow chart of the study

During the study period, a total of 216 cancer patients with nosocomial fungal infections were included in this study (Fig. 1). One hundred thirty-eight were males (64%), and 78 were females (36%). The median age was 65 years old. Among them, 90% of patients had an ECOG-PS of 0–2, and 74% had a TNM stage of III-IV. The common malignancy diagnoses were respiratory tumors (34%), gastrointestinal tumors (24%), and hepatobiliary and pancreatic tumors (24%). Regarding the detailed anti-tumor therapy, 72 patients (33.3%) underwent surgery, 62 patients (29%) received chemotherapy, and 13 patients (6%) received immune checkpoint inhibitors, respectively. Sixty-nine patients (32%) received glucocorticoids within 30 days (Table 1).

**Table 1:** The general characteristics of cancer patients with nosocomial fungal infection

Variable	Overall, N = 216^1^	Survival, N = 159^1^	Death, N = 57^1^	*P*-value^2^
**Demographic data**				
Age (years)	65 (58, 71)	65 (59, 71)	65 (58, 71)	> 0.900
Gender				0.200
male	138 (64%)	98 (62%)	40 (70%)	
female	78 (36%)	61 (38%)	17 (30%)	
Smoking history (Yes)	97 (45%)	66 (42%)	31 (54%)	0.094
**Days of hospitalization(days)**	17 (9, 27)	17 (10, 27)	16 (8, 26)	0.400
**ECOG-performance status**				< 0.001
0,1,2	194 (90%)	153 (96%)	41 (72%)	
3,4	22 (10%)	6 (4.0%)	16 (28%)	
**TNM stage**				0.014
Stage I-II	57 (26%)	49 (31%)	8 (14%)	
Stage III-IV	159 (74%)	110 (69%)	49 (86%)	
**Underlying cancer type**				0.400
Head and neck cancer	7 (3.2%)	6 (3.8%)	1 (1.75%)	
Lung cancer	73 (34%)	50 (31.4%)	23 (40%)	
Esophago-gastrointestinal cancer	35 (16%)	26 (16.3%)	9 (16%)	
Colon and rectal cancer	17 (8.0%)	10 (6.3%)	7 (12%)	
Hepatobiliary and pancreatic cancer	52 (24%)	42 (26.4%)	10 (18%)	
Breast cancer	6 (2.8%)	5 (3.1%)	1 (1.75%)	
Genitourinary cancer	8 (3.6%)	4 (2.5%)	4 (7.0%)	
Gynecological cancer	10 (4.6%)	9 (5.7%)	1 (1.75%)	
Lymphoma	4 (1.9%)	4 (2.5%)	0 (0%)	
Others	4 (1.9%)	3 (2.0%)	1 (1.75%)	
**Distant metastasis**				
Liver metastasis	32 (15%)	26 (16%)	6 (11%)	0.300
Lung metastasis	31 (14%)	18 (11%)	13 (23%)	0.034
Brain metastasis	10 (4.6%)	5 (3.1%)	5 (8.8%)	0.130
Bone metastasis	40 (19%)	24 (15%)	16 (28%)	0.030
Other metastasis	41 (19%)	29 (18%)	12 (21%)	0.600
**CCI score**				0.012
0–3	207 (96%)	156 (98%)	51 (89%)	
> 3	9 (4.0%)	3 (2.0%)	6 (11%)	
**Operation type (within 30 days)**				0.017
Unoperated	144 (67%)	99 (62%)	45 (79%)	
Curative operation	60 (27.7%)	52 (33%)	8 (14%)	
Palliative operation	12 (5.6%)	8 (5.0%)	4 (7.0%)	
**Prior treatment (within 30 days)**				
Chemotherapy	62 (29%)	47 (30%)	15 (26%)	0.600
Radiotherapy	20 (9.3%)	14 (8.8%)	6 (11%)	0.700
Concurrent chemoradiotherapy	11 (5.1%)	8 (5.0%)	3 (5.3%)	> 0.900
Perfusion therapy	11 (5.1%)	10 (6.3%)	1 (1.8%)	0.300
Immunotherapy	13 (6.0%)	7 (4.4%)	6 (11%)	0.110
Targeted therapy	15 (6.9%)	8 (5.0%)	7 (12%)	0.075
Glucocorticoid therapy	69 (32%)	53 (33%)	16 (28%)	0.500
G-CSF usage	47 (22%)	35 (22%)	12 (21%)	0.900

Abbreviations: *ECOG* Eastern Cooperative Oncology Group, *CCI* Charlson Co-morbidity Index score, *G-CSF* granulocyte colony-stimulating factor

^1^n (%); Median (IQR),^2^Pearson’s Chi-squared test; Wilcoxon rank sum test; Fisher’s exact test

### Infection-related features of the patients in the study

In this study, the respiratory tract was the most predominant primary infection site (59.3%), followed by the abdominal cavity (8.8%). *C. Albicans* was the predominant pathogen (68%), followed by other *Candida* species (19%). Two patients (0.8%) were complicated with two or more fungal infections. During hospitalization, 140 patients (65%) received intravenous antifungal therapy. Of these people, 122 patients (56.5%) received triazole antifungal drugs, followed by echinocandin antifungal drugs (5.1%). At the same time, 3.2% of patients received two or more intravenous antifungal drugs. Thirty-four patients (16%) had a history of previously known infection within 30 days before they were diagnosed with a fungal infection. 78 patients (36%) received antibacterial therapy (including empiric antimicrobial therapy) within 30 days before they were diagnosed with a fungal infection. Of all patients, 135 had undergone invasive procedures in the past 30 days before being diagnosed with a fungal infection, with indwelling catheterization being the most common (28%). Furthermore, out of the 216 study participants, 43 (20%) were admitted to the intensive care unit (ICU), and 29 (13%) required mechanical ventilation during their hospital stay. Notably, the overall mortality rate among the study participants was 26.4% (57/216).

### Identification of risk factors for in-hospital death of nosocomial fungal infections

We examined the correlation between the patients’ prognoses and clinical characteristics. The findings revealed significant variability in factors such as ECOG-PS, TNM stage, presence of pulmonary and liver metastases, CCI score, receipt of surgery or chemotherapy within 30 days, as well as laboratory results including platelet count, serum albumin level, serum calcium level, and serum sodium level (*P* < 0.05; Table 1). Meanwhile, there were variations between the two groups in terms of body temperature, antifungal therapy, immunoglobulin therapy, admission to the ICU, mechanical ventilation, and type of sepsis (*P* < 0.05; Table 2). The results of the univariate logistic analysis revealed that several factors were associated with in-hospital death, including ECOG-PS 3–4, TNM stage III-IV, lung metastasis, bone metastasis, radical surgery within 30 days, CCI, admission to the ICU, mechanical ventilation, hypoproteinemia, thrombocytopenia, and hyponatremia. Subsequently, the multivariate analysis identified ECOG-PS 3–4 (OR = 6.08, 95% CI: 2.04–18.12, *P* = 0.001), pulmonary metastases (odds ratio [OR] = 2.76, 95% confidence interval (CI): 1.11–6.85, *P* = 0.029), thrombocytopenia (OR = 2.58, 95% CI: 1.21–5.47, *P* = 0.014), hypoalbuminemia (OR = 2.44, 95% CI: 1.22–4.90, *P* = 0.012), and mechanical ventilation (OR = 2.64, 95% CI: 1.03–6.73, *P* = 0.042) as independent factors influencing in-hospital death in solid-tumor patients with in-hospital fungal infections (Table 3).

**Table 2:** The infection-related characteristics of cancer patients with nosocomial fungal infection

Variable	Overall, N = 216^1^	Survival, N = 159^1^	Death, N = 57^1^	*P*-value^2^
**Primary sites of infection**				0.500
Respiratory tract	128 (59.3%)	93 (58.5%)	35 (61.4%)	
Digestive tract	15 (6.9%)	11 (6.9%)	4 (7.0%)	
Urinary tract	11 (5.1%)	7 (4.4%)	4 (7.0%)	
Thoracic cavity	5 (2.3%)	5 (3.1%)	0 (0%)	
Abdominal cavity	19 (8.8%)	16 (10.1%)	3 (5.3%)	
**Fungi types**				0.200
*Candida albicans*	146 (68%)	114 (71.7%)	32 (56.1%)	
*Mycotoruloides*	41 (19%)	26 (16.4%)	15 (26.3%)	
*Aspergillus flavus*	6 (2.6%)	4 (2.5%)	2 (3.5%)	
*Aspergillus*	18 (8.3%)	11 (6.9%)	7 (12.3%)	
*Penicillium*	1 (0.5%)	1 (0.6%)	0 (0%)	
Coinfection	2 (0.8%)	1 (0.6%)	1 (1.8%)	
Others	2 (0.8%)	2 (1.3%)	0 (0%)	
**Types of antifungal drugs**				0.005
Unantifungal treatment	76 (35.2%)	63 (39.6%)	13 (22.9%)	
Triazole antifungal agent	122 (56.5%)	88 (55.3%)	34 (59.5%)	
Echinocandin antifungal agent	11 (5.1%)	6 (3.8%)	5 (8.8%)	
Combination therapy	7 (3.2%)	2 (1.3%)	5 (8.8%)	
**Length of antifungal treatment (days)**	4 (0, 8)	3 (0, 8)	5 (1, 9)	0.110
**Temperature(≥ 38°C)**	68 (31%)	43 (27%)	25 (44%)	0.019
**Infection history (within 30 days)**	34 (16%)	23 (14%)	11 (19%)	0.400
**Antibiotic usage(within 30 days)**	78 (36%)	52 (33%)	26 (46%)	0.082
**FN history (within 30 days)**	3 (1.4%)	2 (1.3%)	1 (1.8%)	> 0.900
**Invasive procedure (within 30 days)**	135 (62%)	104 (65%)	31 (54%)	0.140
Biliary stent implantation	7 (3.2%)	7 (4.4%)	0 (0%)	0.200
Ureteral stent implantation	3 (1.4%)	2 (1.3%)	1 (1.8%)	> 0.900
Indwelling urinary catheter	60 (28%)	46 (29%)	14 (25%)	0.500
PICC	16 (7.4%)	12 (7.5%)	4 (7.0%)	> 0.900
Infusion port implantation	3 (1.4%)	3 (1.9%)	0 (0%)	0.600
Thoracic puncture catheter drainage	34 (16%)	25 (16%)	9 (16%)	> 0.900
Abdominal catheterization	19 (8.8%)	15 (9.4%)	4 (7.0%)	0.600
Arterial catheterization	5 (2.3%)	1 (0.6%)	4 (7.0%)	0.018
Central venous pressure apparatus	11 (5.1%)	8 (5.0%)	3 (5.3%)	> 0.900
Postoperative drainage	59 (27%)	50 (31%)	9 (16%)	0.023
Indwelling gastric tube	49 (23%)	37 (23%)	12 (21%)	0.700
**Combined with bacterial infection**	54 (25%)	37 (23%)	17 (30%)	0.300
**Immunoglobulin use**	40 (19%)	23 (14%)	17 (30%)	0.010
**ICU admission**	43 (20%)	26 (16%)	17 (30%)	0.029
**Mechanical ventilation**	29 (13%)	14 (8.8%)	15 (26%)	< 0.001
**Cardiac arrest**	12 (5.6%)	0 (0%)	12 (21%)	< 0.001
**Sepsis classification**				< 0.001
None	176 (81.5%)	140 (88.1%)	36 (63.2%)	
Sepsis	21 (9.7%)	15 (9.4%)	6 (10.5%)	
Severe sepsis	5 (2.3%)	3 (1.9%)	2 (3.5%)	
Septic shock	14 (6.5%)	1 (0.6%)	13 (22.8%)	
**Laboratory indexes**				
Hemoglobin(g/L)	103 (90, 120)	106 (93, 120)	98 (85, 115)	0.041
< 110	131 (61%)	94 (59%)	37 (65%)	0.400
Platelet count (×10^9^/L)	176 (111, 252)	197 (132, 266)	117 (58, 210)	< 0.001
< 100	50 (23%)	26 (16%)	24 (42%)	< 0.001
Leucocyte count (×10^9^/L)	8.0 (5.4, 11.2)	8.1 (5.4, 11.1)	7.9 (5.1, 11.9)	0.700
< 4.0	33 (15%)	26 (16%)	7 (12%)	0.500
＞10.0	78 (36%)	37 (23.3%)	35 (61.4%)	0.874
Neutrophils(×10^9^/L)	6.4 (3.7, 9.3)	6.3 (3.5, 9.2)	6.8 (4.3, 10.8)	0.300
Lymphocyte(×10^9^/L)	0.82 (0.52, 1.11)	0.84 (0.52, 1.14)	0.71 (0.48, 1.06)	0.140
Monocyte(×10^9^/L)	0.42 (0.25, 0.71)	0.42 (0.25, 0.72)	0.40 (0.23, 0.68)	0.800
Albumin(g/L)	30.9 (28.2, 35.0)	31.8 (28.8, 36.0)	29.0 (25.8, 31.2)	< 0.001
< 30	90 (42%)	55 (35%)	35 (61%)	< 0.001
Serum calcium(mmol/L)	2.06 (1.95, 2.20)	2.09 (1.98, 2.21)	2.01 (1.89, 2.12)	0.006
< 2.0	144 (67%)	114 (72%)	30 (53%)	0.009
Serum corrected calcium(mmol/L)	2.28 (2.20, 2.38)	2.28 (2.21, 2.38)	2.27 (2.18, 2.40)	0.800
Serum sodium(mmol/L)	138.0(134.0,140.1)	138.4(135.6, 141.0)	135.3(131.0,139.0)	0.020
< 130	61 (28%)	36 (23%)	25 (44%)	0.002

Abbreviations: *PICC* peripherally inserted central catheter, *ICU* Intensive Care Unit

^1^n (%); Median (IQR),^2^Pearson’s Chi-squared test; Wilcoxon rank sum test; Fisher’s exact test

**Table 3:** Logistic regression analysis to identify the influencing factors of in-hospital death in cancer patients with nosocomial fungal infection

Variable		OR (univariable)	OR (multivariable)
ECOG-PS	0,1,2	REF (1.00)	REF (1.00)
	3,4	9.95 (3.66–27.04, *P* < 0.001)	6.08 (2.04–18.12, *P* = 0.001)
TNM stage	I-II	REF (1.00)	
	III-IV	2.73 (1.20–6.19, *P* = 0.016)	
Pulmonary metastasis	Yes	2.31 (1.05–5.10, *P* = 0.037)	2.76 (1.11–6.848, *P* = 0.029)
Bone metastasis	Yes	2.20 (1.07–4.52, *P* = 0.033)	
Operation type	Unoperated	REF (1.00)	
	Radical operation	0.34 (0.15–0.77, *P* = 0.010)	
	Palliative operation	1.10 (0.31–3.84, *P* = 0.881)	
ICU admission	Yes	2.17 (1.07–4.40, *P* = 0.031)	
CCI	≤ 3	REF (1.00)	
	> 3	2.69 (1.13–6.40, *P* = 0.026)	
Platelet count (×10^9^/L)	< 100	3.72 (1.90–7.29, *P* < 0.001)	2.58 (1.21–5.47, *P* = 0.014)
Albumin(g/L)	< 30	3.01 (1.61–5.62, *P* < 0.001)	2.44 (1.22–4.90, *P* = 0.012)
Serum sodium(mmol/L)	< 130	2.67 (1.41–5.07, *P* = 0.003)	
Mechanical ventilation	Yes	3.70 (1.65–8.28, *P* = 0.002)	2.64 (1.03–6.73, *P* = 0.042)

Abbreviations: *OR* odds ratio, *ECOG-PS* Eastern Cooperative Oncology Group Performance Status, *ICU* intensive care u unit, *CCI* Charlson Co-morbidity Index score

### Nomogram establishment and evaluation

**Fig. 2:**
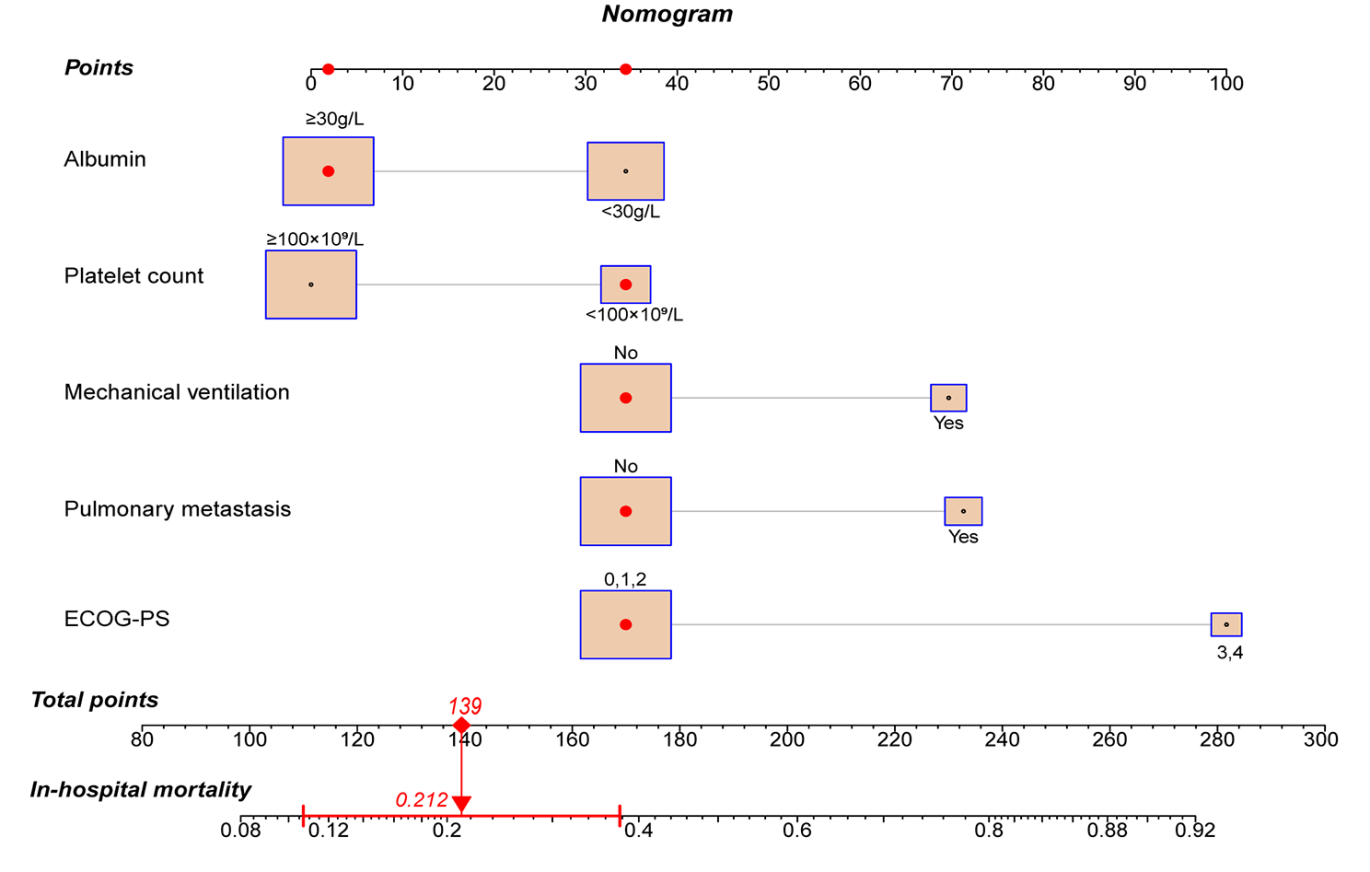
A nomogram to predict the risk of in-hospital death from fungal infections in cancer patients. This patient’s albumin level was 35 g/L, platelet count was 88 × 10^9^/L, without mechanical ventilation, no pulmonary metastasis and ECOG-PS 1. According to the nomogram, we can calculate that the total point for this patient is 139 and its corresponding in-hospital death risk is 21.2%

**Fig. 3:**
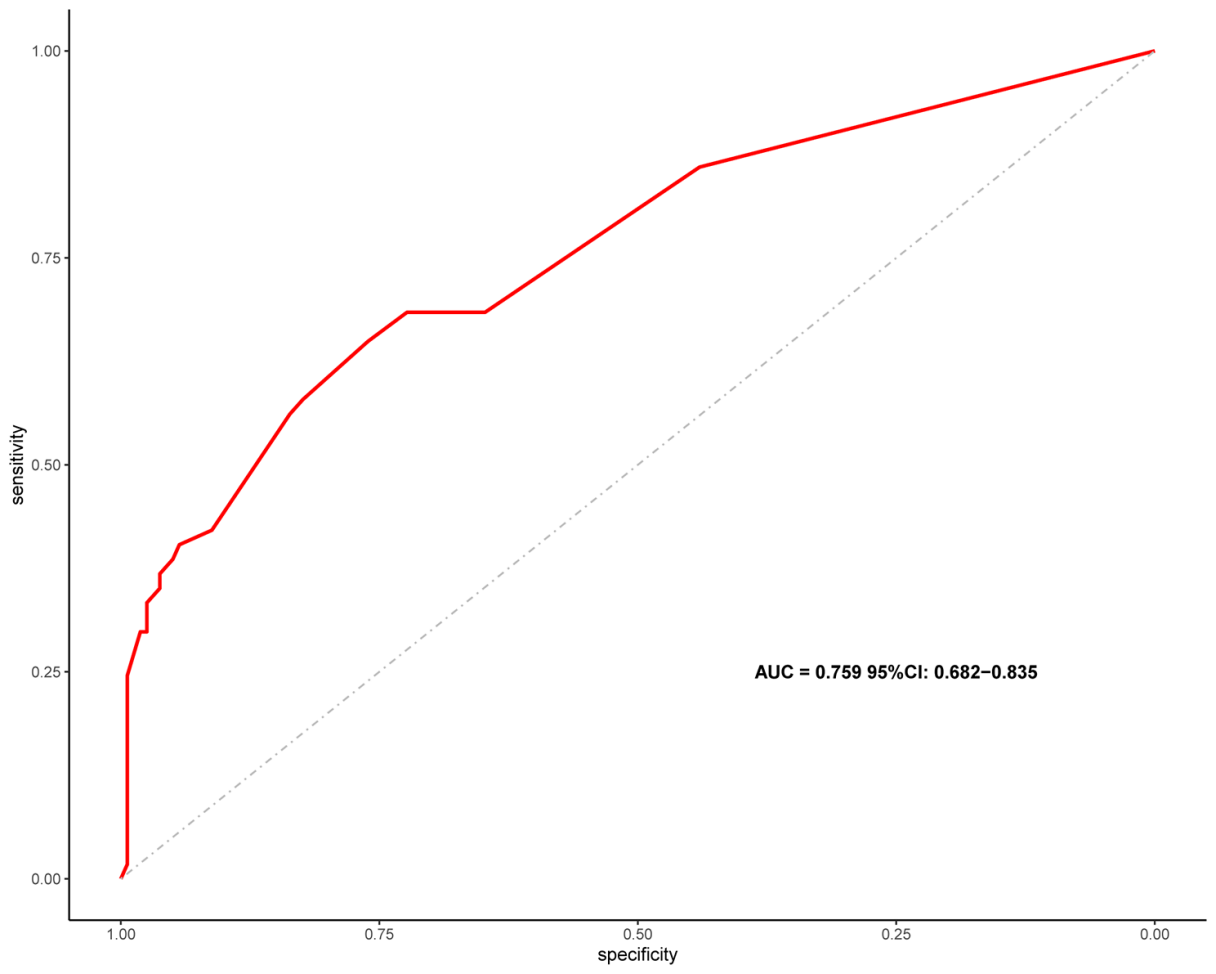
The ROC curve to evaluate the discrimination ability of the nomogram. AUC = 0.759 (95%CI: 0.682–0.835). ROC, receiver operating characteristic curve

**Fig. 4:**
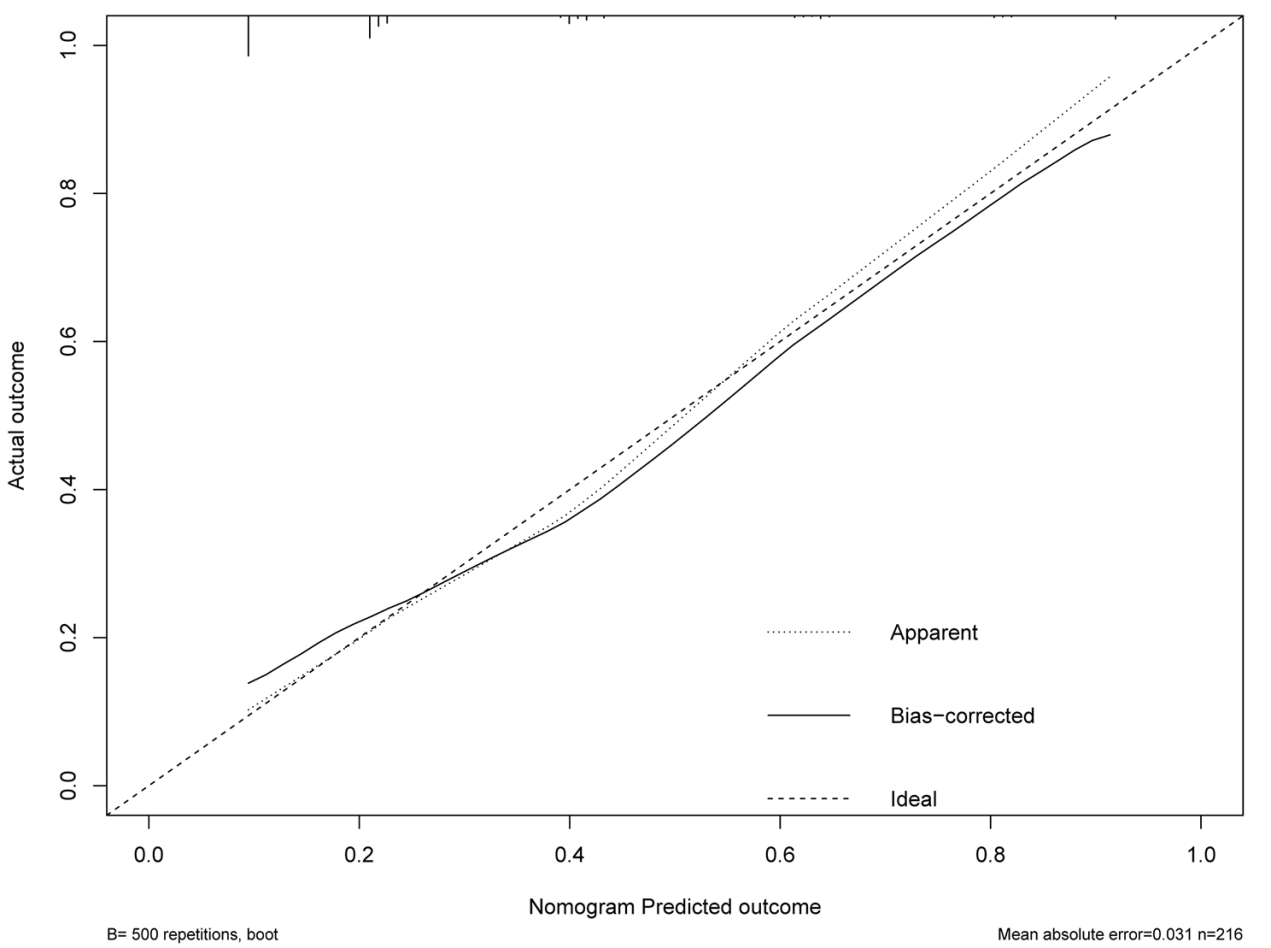
The calibration curve of the nomogram for predicting in-hospital death risk of nosocomial infections caused by fungi in cancer patients

**Fig. 5:**
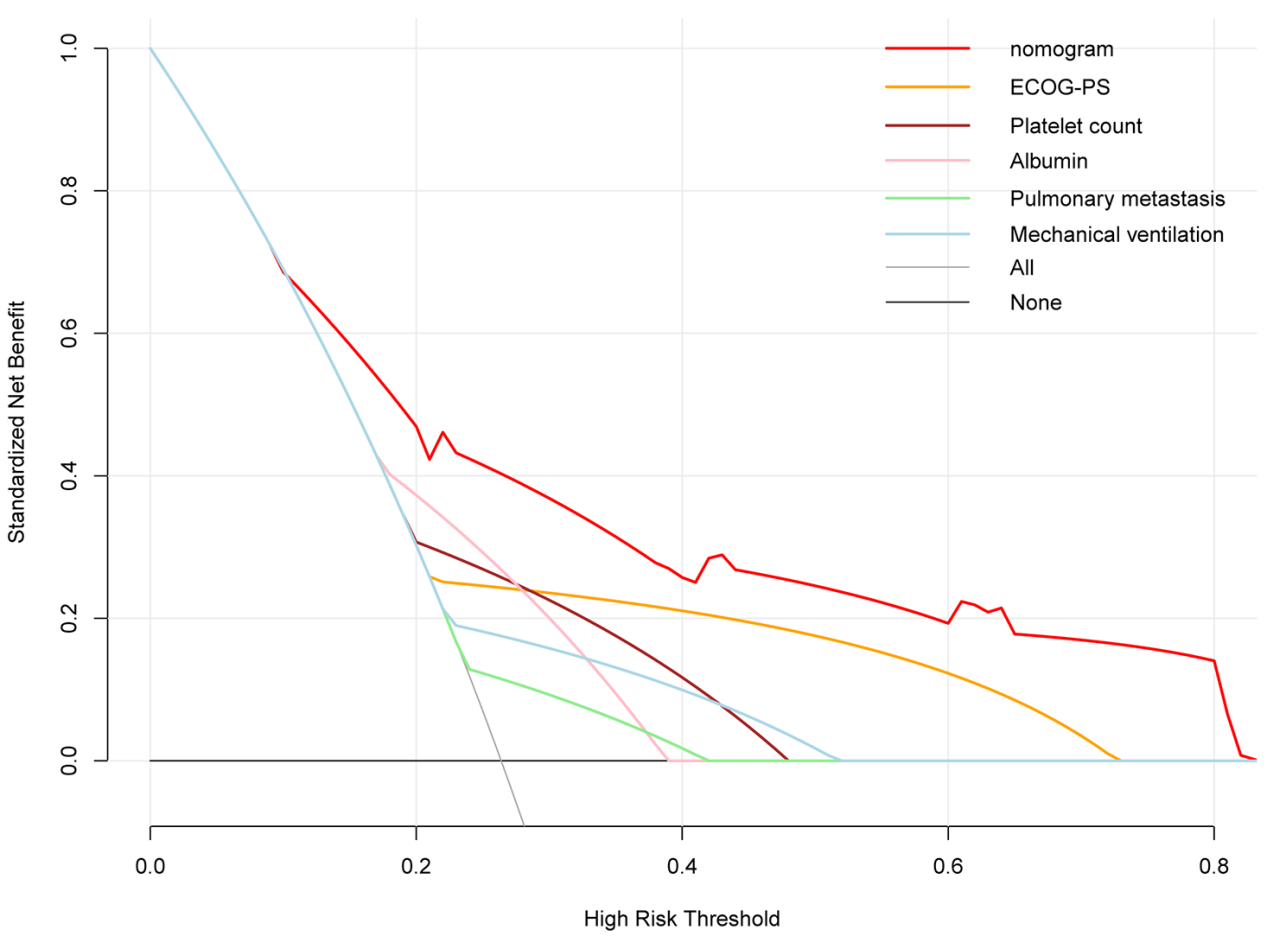
Decision curve analysis of the nomogram for predicting in-hospital death risk of nosocomial infections caused by fungi in cancer patients

Nowadays, nomograms are widely used in clinical medicine research to convert regression models into easily interpretable risk score systems. In this study, we employed a multivariate logistic regression analysis to identify independent predictive factors. Based on these factors, we developed a nomogram **(**Fig. 2**)** to predict the risk of in-hospital death due to nosocomial fungal infection in cancer patients. Based on the contribution of each independent factor to the outcome in the nomogram, clinicians can readily assess the personalized risk of in-hospital death during fungal infection. We used multiple methods to assess the performance of this nomogram, including ROC curve, calibration curve, and DCA. The area under the ROC curve (AUC) of the nomogram was 0.759 (95%CI: 0.682–0.835) (Fig. 3), suggesting an excellent discrimination ability in predicting the nosocomial death risk of patients. Besides, the calibration curve showed a high consistency between the actual in-hospital mortality and the estimated probability through the nomogram (Fig. 4). As it is well-known, ROC curve and calibration curve rely on sensitivity and specificity and may not accurately identify “false positive” and “false negative” events. Hence, we conducted DCA to assess the net clinical benefit of the nomogram. The findings demonstrated that the nomogram consistently provided greater net clinical benefit across the entire range of risk thresholds compared to individual factors alone (Fig. 5). In summary, the developed nomogram serves as a reliable risk classifier for predicting the risk of in-hospital death due to nosocomial fungal infections in patients with solid tumors.

## Discussion

In our study, the prevalence of fungal infections during hospitalization among cancer patients over the 8-year study period was 1.3%, which was found to be lower than that reported in previous studies [26]. It could be attributed by the fact that the incidence of healthcare-related infections varies among different regions. We observed that respiratory tumors accounted for the most common malignancy type that occurs nosocomial fungal infections. On the one hand, according to the newest cancer statistics, respiratory tumors remain the most common malignancies all over the world[27]. On the other hand, emerging evidence has shown that respiratory tumor cells could secrete immunosuppressive factors, which will inhibit the normal natural barrier function of the respiratory tract. Ultimately, lung cancer patients are more susceptible to co-infection compared to other tumor patients due to increased alveolar and bronchial secretions, as well as bronchial mass obstruction [28, 29].

In this study, we found that C. Albicans was the predominant causative pathogen, accounting for 68% of the isolates, followed by other Candida genera (19%). These findings are in line with previous studies [11, 30]. In this retrospective study, 76 patients (36%) received antibacterial therapy within 30 days before the diagnosis of fungal infection, which is the most crucial treatment received in the previous 30 days for this study population. This finding aligns with our standard perspective, suggesting that the use of antibiotics may disrupt the microbial balance and promote fungal overgrowth [31]. Furthermore, within this subpopulation, we observed that 26 participants succumbed to nosocomial fungal infections, representing 46% of all recorded deaths. This highlights the importance of vigilant monitoring by clinicians for patients who experience nosocomial fungal infections, as these individuals are more likely to experience adverse clinical outcomes compared to other subpopulations. A total of 135 patients, representing 62% of the total population, underwent invasive procedures prior to the diagnosis of fungal infection. These procedures, including thoracic or abdominal puncture and central venous catheter (CVC) placement, can result in damage to the mucous membranes of body cavities and the inner walls of blood vessels. This compromised physiological immune barrier renders patients more vulnerable to fungal displacement and colonization, thereby elevating the risk of infection [32].

In the current study, we identified ECOG-PS 3–4, lung metastases, mechanical ventilation, thrombocytopenia, and hypoalbuminemia as independent risk factors for in-hospital mortality due to nosocomial fungal infections in cancer patients. Generally, cancer patients with poor ECOG-PS and distant metastases exhibit restricted physical functioning and a considerable tumor burden, resulting in unfavorable clinical outcomes. The prognostic importance of mechanical ventilation in cancer patients with nosocomial infections during hospitalization has been extensively reported [22, 25, 33, 34]. We found that patients with hypoalbuminemia and thrombocytopenia were associated with higher in-hospital mortality. Serum albumin, a marker of ’patients’ nutritional status, often indicates immunosuppression, malnutrition, and cachexia in individuals with malignancy. The presence of hypoproteinemia in these patients is associated with a poor prognosis and an increased risk of cancer-related deaths [35–37]. Moreover, a growing body of studies have demonstrated that thrombocytopenia is correlated with an unfavorable prognosis in various diseases, including cancer and infections [3, 38].

Simply identifying risk factors for in-hospital mortality of nosocomial fungal infection is insufficient to assist clinicians in making precise and timely decisions. Consequently, we have developed a dependable risk stratification system that incorporates the identified variables to accurately predict the likelihood of in-hospital death in this patient population. Subsequent evaluation demonstrated that our nomogram exhibits satisfactory discrimination ability, calibration ability, and net clinical benefit. Notably, the findings from DCA revealed its significant superiority in terms of net clinical benefits compared to other variables. In summary, the developed nomogram serves as a reliable tool for predicting personalized in-hospital death risk associated with nosocomial fungal infections in cancer patients. To the best of our knowledge, this study represents the first comprehensive investigation of the clinical characteristics, microbiological distribution, and clinical outcomes associated with nosocomial fungal infections among cancer patients in China. Notably, we have also developed a dependable nomogram capable of accurately predicting in-hospital mortality rates for these patients. However, our study had several limitations that should be acknowledged. Firstly, the retrospective design introduced potential biases that were unavoidable. Secondly, we were unable to collect certain variables, such as detailed chemotherapy regimen, radiotherapy dosage, and laboratory results of D-glucan and galactomannan tests, which could have impacted the clinical outcomes of the participants. Thirdly, although the developed nomogram demonstrated excellent predictive power, it is essential to conduct independent external validation in the future to confirm its generalizability. Therefore, well-designed large-scale cohort studies should be undertaken to validate our findings.

## Conclusion

Nosocomial fungal infections are prevalent among cancer patients, with *Candida albicans* being the most frequently isolated causative pathogen. Furthermore, these infections have been linked to adverse clinical outcomes in these individuals. Moreover, we constructed a robust nomogram that could accurately forecasting the risk of in-hospital mortality resulting from nosocomial fungal infections in cancer patients. Implementing meticulous patient management strategies, such as closely monitoring serum albumin levels and platelet counts, administering timely interventions, and providing precise care for individuals with lung metastases and high ECOG-PS scores, could significantly enhance the prognosis of nosocomial fungal infections in this population.

## Data Availability

The datasets used and analyzed during the current study available from the corresponding author on reasonable request.

## Declarations

### Statement

All methods of this study were carried out in accordance with relevant guidelines and regulations.

## Abbreviations

EMR: Electronic Medical Record
FN: Febrile neutropenia
OR: Odds Ratio
CI: Confidence interval
ECOG-PS: Eastern Cooperative Oncology Group-Performance Status
CCI: Charlson Comorbidity Index
G-CSF: Granulocyte Colony-stimulating Factor
ICU: Intensive Care Unit
PICC: Peripherally Inserted Central Catheter
PNI: Prognostic Nutritional Index
NSCLC: Non-small Cell Lung Cancer
ICIs: Immune Checkpoint Inhibitors
